# Effects of Dietary Supplementation of Astaxanthin and Sesamin on Daily Fatigue: A Randomized, Double-Blind, Placebo-Controlled, Two-Way Crossover Study

**DOI:** 10.3390/nu10030281

**Published:** 2018-02-28

**Authors:** Ayano Imai, Yuriko Oda, Naoki Ito, Shinobu Seki, Kiyotaka Nakagawa, Teruo Miyazawa, Fumitaka Ueda

**Affiliations:** 1 Pharmaceutical and Healthcare Research Laboratories, Research and Development Management Headquarters, FUJIFILM Corporation, 577, Ushijima, Kaisei-machi, Ashigarakami-gun, Kanagawa 258-8577, Japan; yuriko.oda@fujifilm.com (Y.O.); naoki.a.ito@fujifilm.com (N.I.); shinobu.seki@fujifilm.com (S.S.); fumitaka.ueda@fujifilm.com (F.U.); 2 Food and Biodynamic Chemistry Laboratory, Graduate School of Agricultural Science, Tohoku University, Sendai 980-0845, Japan; nkgw@m.tohoku.ac.jp (K.N.); miyazawa@m.tohoku.ac.jp (T.M.); 3 New Industry Creation Hatchery Center (NICHe), Tohoku University, Sendai 980-8579, Japan

**Keywords:** astaxanthin, sesame seed extract, sesamin, fatigue, visual analogue scale, phosphatidylcholine hydroperoxide

## Abstract

Severe fatigue can negatively affect quality of life, and oxidative stress may play a role in its mechanism. The aim of this study was to evaluate the effect of dietary supplementation of astaxanthin and sesamin (AS), strong food-derived antioxidants, on fatigue. Twenty-four healthy volunteers were supplemented with AS and placebo, each for four weeks. After each supplementation period, participants underwent tasks inducing mental and physical fatigue (visual display terminal task and ergometer task, respectively). Subjective fatigue was evaluated using a visual analogue scale during and after the mental and physical tasks, and daily subjective fatigue was evaluated by the Chalder fatigue questionnaire. Secondary outcomes included other subjective feelings, work efficiency, autonomic nerve activity, levels of an oxidative stress marker (plasma phosphatidylcholine hydroperoxide (PCOOH)) and safety. AS supplementation was associated with significantly improved recovery from mental fatigue compared with placebo. Increased PCOOH levels during mental and physical tasks were attenuated by AS supplementation. No differences between AS and placebo were detected in secondary outcomes, and no adverse effects of AS supplementation were observed. In conclusion, AS supplementation may be a candidate to promote recovery from mental fatigue which is experienced by many healthy people.

## 1. Introduction

Fatigue in healthy people is an important biological alarm system that acts as a cue to rest. However, long-lasting or extensive fatigue can have a negative effect on quality of life.

Oxidative stress is involved in the mechanism of exercise-induced fatigue [1] and mental stress [2] in healthy humans, as well as the pathophysiology of prolonged fatigue (chronic fatigue syndrome) [3,4,5]. One biological marker of oxidative stress is phosphatidylcholine hydroperoxide (PCOOH), which is the major phospholipid hydroperoxide in plasma [6]. Because phospholipids, including phosphatidylcholine, are a major component of cell membranes, extensive lipid peroxidation can lead to loss of fluidity, falls in membrane potential and eventual rupture, leading to release of cell and organelle contents [7].

Astaxanthin is a red oceanic carotenoid that is found in shellfish, algae and fish. Its strong antioxidative activity has been reported by several groups [8,9]. In particular, its singlet oxygen quenching ability was reported to be approximately 1000 times more effective than coenzyme Q10 [8]. Various biological activities have been reported for astaxanthin, such as anti-inflammatory [10], skin moisture-improving [11], immune-stimulatory [12] and neuroprotective activities [13]. For example, astaxanthin attenuates amyloid β induced erythrocyte damage in vitro and in vivo [14]. Dietary intake of astaxanthin has also been shown to improve erythrocyte antioxidant status and decrease phospholipid peroxide levels in humans [6].

Sesame is an oleaginous seed which has traditionally been considered to be a health food in Japan and other Asian countries. Sesamin is one of the lignans abundantly present in sesame seed. Sesame seed extracts display antioxidant activity in the β-carotene/linoleic acid system, which evaluates inhibition of the formation of free radicals generated during the peroxidation of linoleic acid [15]. Their antioxidative activities can be attributed to the presence of antioxidant lignans such as sesamin [16].

Several studies have tested the effects of astaxanthin or sesame lignan separately on fatigue. Hongo et al., reported that astaxanthin reduced daily fatigue stemming from mental and physical causes [17]. Takemoto et al., evaluated the effects of supplements including sesame lignans and vitamin E on subjective outcomes such as fatigue, sleep and physical appearance [18]. However, the combination of these two components has never been studied for anti-fatigue activity. The objective of this study is to investigate the effect of a mixed supplement containing astaxanthin and sesamin (AS) on daily fatigue. We evaluated subjective fatigue using markers of daily fatigue (visual analog scale (VAS) for fatigue and the Chalder fatigue questionnaire (CFQ)).

## 2. Materials and Methods

### 2.1. Study Design

**Figure 1:**
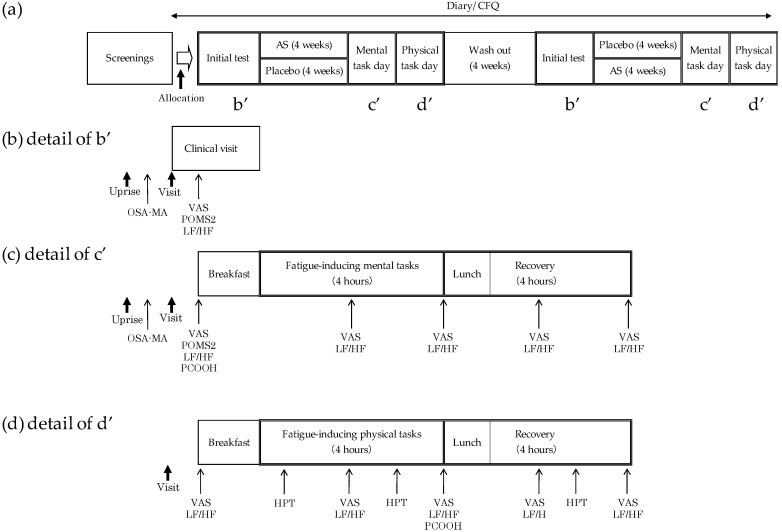
Study design and major evaluation items. (**a**) Study overview; (**b**) schedule of initial test day; (**c**) schedule of mental task day; (**d**) schedule of physical task day.

This study was performed in a randomized, double-blind, placebo-controlled, two-way crossover design as shown in Figure 1a. We chose crossover design to minimize confounding covariates arising from differences in subjective fatigue score. An equal number of participants was allocated to two groups. The study was carried out from August 27 to 11 December 2016. An adequate wash out period was determined as four weeks between first and second test periods, which is >5 half-lives of astaxanthin and sesamin. The half-lives of astaxanthin and sesamin are reported as between 12 and 22 h [19,20] and about 4.7 h, respectively [21]. Participants were instructed to maintain their regular diet and not to consume an excessive amount of food and alcoholic beverages. Consuming anti-fatigue dietary supplements and/or excessive amounts of beverages and food containing astaxanthin or sesamin was also prohibited. The study protocol complied with the principals of the Declaration of Helsinki and was approved by the institutional review board at Fukuda Clinic (approval number: IRB-20160618-1). The protocol was registered at the UMIN-CTR (#UMIN000023757). The protocol was not changed from the time of setup. This study was conducted in Osaka, Japan.

The primary outcomes were subjective fatigue score, assessed by VAS (which is recommended in the guideline published by the Japanese Society of Fatigue Science [22]) and CFQ [23]. The other outcomes were work efficiency, mood state (Japanese version of the Profile of Mood States 2 scale (POMS2)) [24], Ogri-Shirakawa-Azumi sleep inventory MA version (OSA-MA) [25], autonomic nerve activity, oxidative stress marker and safety.

### 2.2. Participants

The study group comprised 24 healthy volunteers. The number of participants was determined in reference to studies that evaluated anti-fatigue effects of food ingredients, such as antioxidative chicken breast extract [26], caffeine and D-ribose [27]. Participants were allocated randomly into two groups by an independent controller (Statcom Co., Ltd., Tokyo, Japan) using SAS 9.3 (SAS Institute Inc., Wallisellen, Switzerland). The allocation was concealed from participants, practitioners and clinicians by the independent controller. The inclusion criteria were (1) healthy males and females aged from 30 to 60 years old (bounds included); (2) subjects with total CFQ scores ≥ 21; (3) subjects able to fully understand the content of the study and its objective and provide written informed consent. Exclusion criteria were (1) subjects receiving medical treatment for serious renal, hepatic, cardiovascular, respiratory, endocrine or metabolic disorders or having a medical history of these disorders; (2) subjects with chronic fatigue syndrome or deemed by the investigator to have severe fatigue (such as idiopathic chronic fatigue); (3) subjects allergic to gelatin; (4) subjects with a medical history of chest pain or syncope; (5) subjects with an abnormality in their electrocardiogram; (6) subjects with subjective symptoms of low back pain, arthralgia, lumbar hernia, disease of lower limbs or palpitation; (7) subjects regularly taking medication or quasi-drugs which may be associated with recovery from fatigue or nutritional support for physical fatigue; (8) subjects regularly consuming processed food containing ingredients known to attenuate the sensation of fatigue; (9) subjects regularly consuming food containing large amounts of carotenoid; (10) subjects who donated or lost more than 200 mL of blood within 1 month, or more than 400 mL within 3 months prior to the start of the present study; (11) subjects who took part in another clinical study within 3 months prior to the start of the present study or who were currently taking part in another clinical study; (12) female subjects who were pregnant or lactating, or intending to become pregnant during the study and (13) subjects deemed to be unsuitable by the investigator.

### 2.3. Screenings and Initial Test

Participants were recruited from a volunteer databank owned by the contract research organization between 19 June and 6 August 2016. All participants signed written informed consent prior to enrollment. First-order screening was conducted in terms of blood pressure, pulse rate, height, body weight, body temperature, blood test, resting electrocardiogram, VAS for fatigue [22], face scale [28], CFQ [23], autonomic nerve activity [29], current medical condition and medical history. Participants who satisfied the inclusion criteria and exclusion criteria were selected for the second-order screening, which included anaerobic threshold measurement, an ergometer cycle task, blood testing, VAS for fatigue, blood pressure, pulse rate, body weight, body temperature and medical interview. The initial test was conducted before supplementation (Figure 1b).

### 2.4. Fatigue-Inducing Tasks

On the mental task day, participants were given a total of 4 h of visual display terminal (VDT)-based mental tasks followed by 4 h of recovery periods. The task consisted of four sets of 30 min of the 2-back test (simple working memory task) [30] and four sets of 30 min of the advanced trail making test (ATMT) (selective attention and spatial working memory task) [31] (Figure 1c). Fatigue-inducing physical tasks were conducted on the second screening day and the second clinic visit day. An ergometer (Aerobike 75XL-II; Konami Sports Club Co., Ltd., Tokyo, Japan) cycle task at a target heart rate was performed for 4 h. The individual workload was determined to at 80% of the anaerobic threshold heart rate measured by the incremental load test using a bicycle ergometer on the second screening day. The ergometer task was followed by a 4-h recovery period (Figure 1d).

### 2.5. Supplements

Softgel capsules, each containing 3 mg of astaxanthin derived from *Haematococcus pluvialis* (ASTOTS, FUJIFILM) and 5 mg of sesamin derived from *Sesamum indicum* L., were given as supplements to participants. Placebo capsules were identical to AS capsules regarding shape, color and taste, which was confirmed by the institutional review board of Fukuda Clinic. Subjects were instructed to take 2 capsules after breakfast every day. On test days, subjects were instructed to take 2 capsules between breakfast and either mental or physical tasks.

### 2.6. Primary and Secondary Outcomes

Subjective fatigue was measured by the VAS on the initial test day and task days. On the initial test day, the VAS was completed once after the clinical visit. On task days, the VAS was completed five times: before the task, 2 and 4 h into the task and 2 and 4 h into the recovery period. A weekly web questionnaire using the CFQ [23] was also completed every Wednesday, starting from 10 days prior to the initial test.

Work efficiency during mental and physical tasks was assessed by the C task of the ATMT [31] and the 10-s high power test (HPT) [32], respectively. On the physical task day, the 10-s HPT was conducted 0.5 and 3.5 h into the physical task and 3.5 h into the recovery period. 

The POMS2 [24] and OSA-MA [25] were used to assess other subjective feelings before and after four weeks of supplementation.

Autonomic nerve activity was assessed by frequency domain analyses of the a–a wave intervals of accelerated plethysmography using ARTETT (U-MEDICA, Inc. Co., Osaka, Japan) [33]. The low-frequency (LF) power was calculated as the power within the frequency range of 0.04 to 0.15 Hz, and the high-frequency (HF) power was calculated as that within the frequency range of 0.15 to 0.4 Hz. The ratio of LF to HF power (LF/HF) was used as an indicator of sympathetic/parasympathetic balance [34]. On the initial test day, LF/HF was measured once after the clinical visit. On task days, LF/HF was measured five times: before the task, 2 and 4 h into the task and 2 and 4 h into the recovery period.

Blood tests to determine oxidative stress marker, astaxanthin content and safety were carried out before and after both mental and physical tasks. As a marker of oxidative stress, plasma PCOOH was evaluated using the following method. Plasma was subjected to the Folch method [35] followed by the solid phase extraction to remove neutral lipid [36]. The obtained phospholipid partition was dissolved in methanol and subjected to following analysis. PCOOH detection was performed by high-performance liquid chromatography (HPLC) with chemiluminescence detection (CLA-FL2, Tohoku Electronic Industries, Miyagi, Japan) [6]. A Finepak SIL-NH2-5 (4.6 mmID × 250 mm (JASCO)) column was used with 2-propanol/methanol/water (135:45:20, *v*/*v*/*v*) as a mobile phase. As a hydroperoxide-specific post-column chemiluminescence reagent, 50 mM-borate buffer (pH 10), containing 2 μM luminol and cytochrome *c* (Sigma-Aldrich, St. Louis, MO, USA, type VI) was used. For the plasma astaxanthin extraction, an aliquot of 500 μL of plasma was mixed with 500 μL of water, 1 mL of ethanol, and 500 μL of 4 μM-ethanolic echinenon. It was then mixed with hexane/dichloromethane (2:2, *v*/*v*, including 0.02% butylated hydroxytoluene) and the supernatant was collected. The collected supernatant was dried under nitrogen gas. The residue was dissolved in methanol/methyl tert-butyl ether (2:3, *v*/*v*, including 0.2% butylated hydroxytoluene) and subjected for analysis. Plasma astaxanthin concentration was evaluated by ultraviolet-HPLC at 463 nm. HPLC conditions were used as described previously [6]. General biochemical examination of blood and hematologic test were performed for safety evaluation.

Subjects were instructed to keep a daily diary every day of their lifestyle.

### 2.7. Statistical Analysis

All data were presented as the means ± S.D. The difference between AS and placebo supplementation was analyzed using 2-way repeated-measures ANOVA with supplementation group and time point as the main factors, and supplementation/time interaction effect when data were parametric and paired and using 2-way factorial ANOVA when data were parametric and unpaired. When comparing within one factor, paired *t* test was used. The Wilcoxon signed-rank test was performed when data were not parametric. Statistical analyses were performed with SPSS Ver.22.0 (IBM Japan, Ltd., Tokyo, Japan). Probabilities less than 5% (*, *p* < 0.05) were considered to be statistically significant. Correlation between the difference in VAS for fatigue score and astaxanthin content in plasma was analyzed by Spearman rank correction analysis. A generalized linear model was used to assess the adequacy of the crossover test.

## 3. Results

### 3.1. Participants

**Figure 2:**
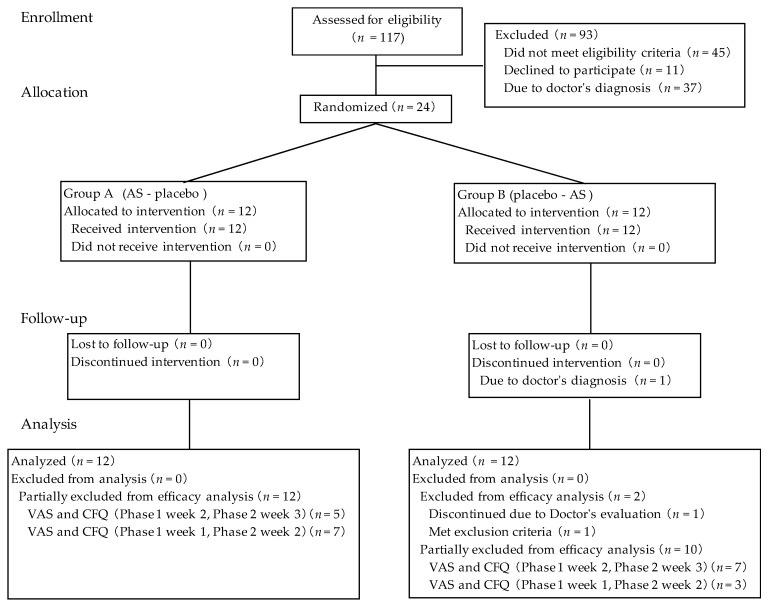
Flow diagram of participants.

The flow diagram in Figure 2 provides an overview of all included and excluded participants. Participants who could endure the fatigue-inducing tasks were included after second-order screening. A total of 24 participants were qualified for allocation after two screenings, and 23 participants completed the study. One participant discontinued after the washout period due to abnormal blood test values which were unrelated to the intake of the test supplement according to the study doctor. During the physical task, workload was reduced in one participant from 45 watt to 34 watt due to the study doctor’s decision based on the increase in heart rate. The variation of heart rate during the physical task was from 70 ± 9 for the initial time point to 82 ± 10 at the end of 4 h physical task. No statistically significant differences in baseline characteristics were observed between the two groups in terms of age, sex, body mass index, fatigue score (CFQ and VAS) at first-order screening (Table 1) and workload during the physical task at second-order screening. For efficacy evaluation, 22 participants were included in the per-protocol efficacy analysis; one participant who dropped out of the study and one participant who was considered to meet exclusion criterion 2 (severe fatigue) due to shingles were excluded from efficacy analysis. For the web questionnaire, assessments completed on the Japanese national holiday (23 November 2016) and during Silver Week, a string of Japanese national holidays (21 September 2016), were excluded from statistical analysis. Ingestion rates of the AS and placebo periods were 99.6 ± 1.5% and 98.6 ± 3.5%, respectively. There was no significant difference between the two test conditions in ingestion rate.

### 3.2. Subjective Fatigue Score on Mental and Physical Task Days

The order effect and the period effect in the crossover design were determined for the VAS at all test days (initial test day, mental task day and physical task day) for both all subjects and eligible subjects. Neither a significant order effect nor period effect were found. Therefore, this study was considered adequate as a crossover study.

**Figure 3:**
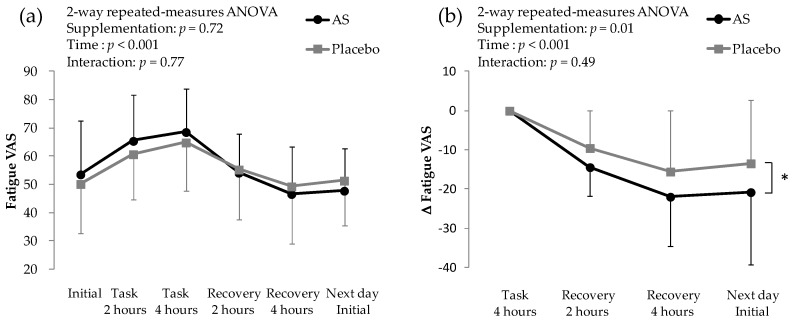
Subjective fatigue evaluated with VAS on mental task day. (**a**) VAS scores on mental task day; (**b**) Difference in VAS scores in recovery period. * *p* < 0.05 between AS and placebo by 2-way repeated-measures ANOVA. Data are presented as the means ± S.D.

On the mental task day, the 2-way repeated-measures ANOVA revealed no interaction, and showed significant time factor, indicating that the subjective fatigue changed along with task (Figure 3a). There was no significant difference between AS and placebo, when task period and recovery period were combinedly analyzed. In the difference from score at the end of the mental task, there was a significant difference between AS and placebo (Figure 3b). VAS scores during the physical task were assessed on the day after mental task. The difference between initial VAS score on the physical task day and the mental task day was statistically significant between AS and placebo supplementations; this was considered to be a carry-over effect. Therefore, VAS scores on the physical task day were considered not valid.

VAS scores were also assessed before and after four weeks of supplementation. The scores before and after four weeks of supplementation were 51.4 ± 16.4 and 53.6 ± 18.8, respectively, for AS supplementation and 49.9 ± 19.1 and 50.2 ± 17.6, respectively, for placebo supplementation. Significant differences between AS and placebo supplementation were not observed.

### 3.3. Fatigue Questionnaires Using CFQ

CFQ scores were assessed every Wednesday via web questionnaires. A 2-way repeated-measures ANOVA revealed that the interaction and the effect of supplementation was not significant. There was a significant difference in time (*p* = 0.02). About the difference from the initial score, the interaction and the effect of supplementation was not significant. There was a significant difference in time (*p* < 0.01).

### 3.4. Secondary Outcomes

For autonomic nervous function, spectral analysis of a–a interval of accelerated plethysmography was performed both before and after four weeks of supplementation and during and after both the mental and physical tasks. LF/HF values before supplementation and after supplementation and the difference between these were 1.867 ± 2.606, 1.654 ± 1.905 and −0.213 ± 1.754, respectively, for AS supplementation and 1.688 ± 1.426, 1.471 ± 1.425 and −0.217 ± 1.931, respectively, for placebo supplementation. There were no significant differences between AS and placebo supplementation.

Work efficiency during the mental task was measured by the C task of the ATMT. No significant differences were observed between AS and placebo supplementation in both numbers of errors and average response time (data not shown).

Work efficiency during the physical task was measured by the 10-s HPT 0.5 and 3.5 h into the physical task and 3.5 h into the recovery period. Results for AS supplementation were 54.5 ± 21.7 revolutions per minute (rpm), 55.2 ± 19.5 rpm and 56.5 ± 18.8 rpm, respectively. Results for placebo supplementation were 58.3 ± 22.0 rpm, 57.3 ± 20.3 rpm and 59.2 ± 20.7 rpm, respectively. There were no significant differences between AS and placebo supplementation.

The POMS2 and OSA-MA were performed for other subjective feeling assessments. The total mood disturbance in POMS2 was not significantly different between AS and placebo supplementation. OSA-MA evaluation of all five factors was not significant between AS and placebo either before or after four weeks of supplementation, and the change between the two time points was also not different (data not shown).

### 3.5. Blood Test

**Figure 4:**
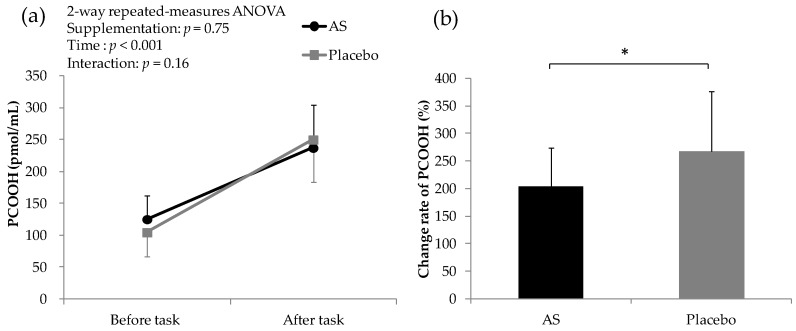
Concentration of PCOOH before and after fatigue-inducing tasks. (**a**) Change in PCOOH before and after task; (**b**) Change rate of PCOOH. * *p* < 0.05 between groups by paired *t* test. Data are presented as the means ± S.D.

PCOOH concentrations are shown in Figure 4. The plasma astaxanthin level after four weeks of AS supplementation was 208 ± 87 pmol/mL, while that after placebo supplementation was not detectable. During fatigue-inducing tasks, plasma PCOOH levels increased after both AS and placebo supplementation. There was no significant difference between AS and placebo supplementation in the raw data. However, the rate of change of plasma PCOOH levels after AS supplementation was significantly lower than that after placebo supplementation.

The correlation between differences in VAS and plasma astaxanthin concentration was analyzed after AS supplementation for the time point at which a significant difference between the AS group and placebo group was observed. Correlation coefficient between plasma astaxanthin content and the difference in VAS score (recovery 2 h-task 4 h) was −0.451 (*p* < 0.05), and the difference in VAS score (recovery 4 h-task 4 h) was −0.502 (*p* < 0.05).

### 3.6. Safety Evaluation

Among the parameters assessed in general biochemical examinations, several statistically significant changes were observed. However, these were within normal limits of daily variation and were not considered to be due to AS or placebo supplementation by the study doctor. No adverse effects associated with the supplementation were observed.

## 4. Discussion

We evaluated the effect of AS on fatigue in healthy participants aged between 30 and 60. Our results suggest that AS promoted recovery from computer-based mental tasks in subjective fatigue. The subjective fatigue score increased after 4 h of the mental task, then decreased throughout the recovery period after both AS and placebo supplementation. Participants recovered more promptly from VDT-induced mental fatigue (involving a simple working memory task using the two-back test and selective attention and spatial working memory tasks using the ATMT) after four weeks of AS supplementation than after placebo supplementation. A relationship between VDT operation and psychological stress has been shown [37]. The results of the present study can be generalized to daily fatigue and psychological stress, because the employed tasks were similar to daily VDT operation. The effect of AS on mental status can be extended to brain function-related symptoms, such as depression.

Correlation between plasma astaxanthin concentration and changes in VAS suggest that this anti-fatigue effect of AS might be associated with the effect of astaxanthin. Astaxanthin can cross the blood–brain barrier and protect the brain from acute injury and chronic neurodegeneration [38]. The neuroprotective properties of astaxanthin are derived from its antioxidative, anti-apoptotic and anti-inflammatory effects [39,40]. Antioxidative and anti-apoptotic activity might be attributable to the upregulated expression of endogenous superoxide dismutase and catalase [41]. An anti-inflammatory mechanism might also be involved in the anti-fatigue effect of astaxanthin, because chicken essence and its dipeptides anserine and carnosine are known to have both anti-inflammatory and anti-fatigue effects [42,43]. The link between inflammation and chronic fatigue syndrome has also been reported [44]. Astaxanthin might therefore be active in the brain under conditions of mental stress to promote recovery from fatigue.

There are several reports of the effects of astaxanthin or sesame-derived compounds after physical exertion [45,46,47]. Astaxanthin has been reported to improve endurance capacity [45] and fat utilization during exercise via CPT-I activation, leading to improved endurance [46]. Sesamin may enhance the degradation of lipid peroxides in the liver, resulting in a strong protective effect against exercise-induced plasma lipid peroxidation [47]. In this study, the effect of AS on physical fatigue could not be evaluated because a carry-over effect from the mental task day was observed. Further investigation is needed to assess the anti-fatigue effect of AS on physical fatigue.

Sesame lignans containing sesamin have been reported to be pro-antioxidant, indirectly acting as an antioxidant via its metabolites [48]. The supplementation of sesame lignans with vitamin E significantly improved subjective fatigue-related status and antioxidative capacity, particularly in middle-aged and elderly people experiencing feelings of daily fatigue [18]. The antioxidative [48,49], anti-inflammatory and neuroprotective properties of sesamin may contribute to the observed effect of AS. Sesamin exhibited anti-inflammatory activity by inhibiting delta 5-desaturase, which plays an important role in the production of pro-inflammatory mediators [50]. Excess generation of nitric oxide in lipopolysaccharide-stimulated BV2 microglial cells was significantly attenuated by sesamin [51]. A neuroprotective effect was also exhibited in gerbil brain in cerebral ischemia [52]. Sesamin was absorbed efficiently and distributed throughout the whole body, especially in the liver and kidney in the form of its metabolites [21]. The difference between astaxanthin and sesamin in distribution throughout the body may contribute to the anti-fatigue effect of AS. Further analysis is needed to evaluate the individual effects of sesamin and astaxanthin.

There are several oxidative stress markers including 8-hydroxy-2′-deoxyguanosine, isoprostane and PCOOH. In this study, we chose PCOOH as an oxidative stress marker, since both astaxanthin and sesamin are lipophilic compounds. PCOOH levels reflect the amount of oxidative stress in vivo, as PCOOH is the primary oxidation product of phospholipids [6]. The relationship between oxidative stress and fatigue has been previously explored [1,2,3,4,5], but most of these investigations targeted people with chronic fatigue syndrome. Consistent with previous research, we found that intensive mental and physical tasks increased plasma PCOOH in healthy humans, and AS suppressed the mental and physical task-induced PCOOH increase. Our results suggest that the anti-fatigue effect of AS may be mediated via reduction of oxidative stress. However, further studies to analyze other oxidative stress markers will be needed to reinforce the link between oxidative stress and daily fatigue in healthy humans.

There were several limitations of this study. Sample size was limited, and a per protocol analysis was performed. A carry-over effect of AS was observed due to mental and physical tasks being performed on two consecutive days. Therefore, we could not assess the effect of AS on physical task-induced fatigue. The concentration of sesamin in plasma was not evaluated; hence, the relationship between subjective fatigue and sesamin is unknown. PCOOH was not measured separately after each mental and physical task. Because only continuous ingestion of AS was evaluated in this study, it is not possible to determine whether the observed effects were due to continuous ingestion or a single ingestion.

## 5. Conclusions

As a result of this four-week randomized, double-blind, placebo-controlled, two-way crossover study, AS supplementation exhibited the effect of promoting recovery from VDT-induced mental fatigue. The increase in PCOOH during mental and physical tasks was attenuated by AS supplementation. Thus, antioxidative activity exhibited by AS could be a possible mechanism for its anti-fatigue effect. Our results suggest the novel possibility that supplementation with AS may reduce subjective fatigue in healthy subjects. The safety of a four-week AS supplementation period was also confirmed.

## Figures and Tables

**Table 1:** Baseline characteristics ^1^.

									VAS Scores on Screening Day		
	Phase 1	Phase 2	*n*	Male	Female	Age	BMI (kg/m^2^)	CFQ Score	Initial	After Physical Task (4 h)	After Recovery (4 h)
Group A	AS	Placebo	12	6	6	44.8 ± 7.2	21.8 ± 2.4	25.1 ± 2.4	36.3 ± 16.2	63.0 ± 16.2	50.3 ± 19.1
Group B	Placebo	AS	10	5	5	43.0 ± 8.6	21.7 ± 2.4	25.4 ± 4.6	36.1 ± 20.0	66.1 ± 10.0	51.6 ± 14.8

^1^ Data are presented as the means ± S.D.
